# A single amino acid mutation in VP1 of coxsackievirus A6 determining efficiency of VP0 cleavage and proliferation

**DOI:** 10.1128/jvi.00128-25

**Published:** 2025-05-14

**Authors:** Yihao Sun, ShaSha Qian, Yaxin Du, Jiahui Wu, Hadireya Rehemutula, Shengli Meng, Zejun Wang, Jing Guo, Shuo Shen

**Affiliations:** 1 Wuhan Institute of Biological Products Co. Ltd. 155181 , Wuhan, People's Republic of China; University of Michigan Medical School, Ann Arbor, Michigan, USA

**Keywords:** CV-A6, VP0 cleavage regulation, proliferation, virulence

## Abstract

Coxsackievirus A6 (CV-A6) has emerged as a major pathogen associated with hand, foot, and mouth disease (HFMD), capable of infecting both children and adults. However, currently, there is no effective vaccine to prevent HFMD caused by non-EV-A71 enteroviruses. In this study, a pair of CV-A6 strains was selected from a rhabdomyosarcoma (RD)-isolated and Vero-adapted stock with a difference of 7 nucleotides in their genomes, resulting in three amino acid mutations in the structural proteins. Distinct differences in propagation, virulence in cells, and plaque size were observed. A series of single-site mutants was constructed, and a single mutation in VP1-143 was mapped to associate with phenotype changes. The mutation from glycine to arginine at VP1-143 dramatically increased infectivity but decreased virulence, growth rate, and plaque size. Furthermore, the experiments using both purified whole virus and full particle (FP) demonstrated that glycine-to-arginine mutation increased VP0 cleavage efficiency because of decreased VP0/VP2 ratio. The decrease in VP0 cleavage efficiency led to the accumulation of non-infectious provirion. The efficiency of virus transmission between cells determined the rates of viral RNA (vRNA) and protein synthesis and was related to fast-slow growth and virulence phenotypes. In addition, the data indicated that the mutation did not affect the encapsidation of the genomic RNA, and the ratio of empty and full particles was unchanged. The results are important for understanding the mechanism of VP0 cleavage regulation and are relevant to developing vaccines and therapeutic reagents against CV-A6 infection and diseases.

**IMPORTANCE:**

CV-A6 is a major pathogen in the context of HFMD. The cost of treatment and hospitalization of children with HFMD may have a considerable financial impact on the families of patients. CV-A6 is a member of picornaviruses and forms infectious virion through maturation cleavage of VP0 into VP4 and VP2. Although it is well accepted that the autocatalytic process involves viral RNA, the detailed mechanism remains unclear. In this study, residues in VP1-143 were demonstrated to regulate the efficiency of VP0 cleavage and affect the ratio of provirion and virion. Glycine-to-arginine mutation was tolerant, not abolished, but affected the efficiency of VP0 cleavage. The results support a theory that residue mutations on a structural protein of a serotype/genotype within enteroviruses, not well-conserved across picornaviruses and far away from the VP0 cleavage site on the outside surface, regulate the efficiency of VP0 cleavage and render phenotypically different strains.

## INTRODUCTION

Coxsackievirus A6 (CV-A6) is a member of species A in the genus Enterovirus within the family Picornaviridae and is one of the primary pathogens responsible for hand, foot, and mouth disease (HFMD) (1). HFMD is a prevalent infectious illness among children, with numerous outbreaks documented globally (2, 3). Infection with CV-A6 can manifest in a variety of atypical clinical symptoms similar to herpes pharyngitis, onychomycosis, epididymitis, and even in severe cases, such as aseptic meningitis or cerebrospinal meningitis (4). Unlike the typical presentation of HFMD, CV-A6 can infect not only infants and young children but also adults (5). Vaccines against HFMD caused by enterovirus A71 (EV-A71) are currently available on the market. However, due to the lack of cross-protection of neutralizing antibodies between different enterovirus serotypes (6), the number of HFMD cases caused by CV-A6 has increased dramatically in recent years, becoming a major pathogen in HFMD outbreaks (7–10).

Enteroviruses are positive-sense single-stranded RNA viruses with a genome approximately 7.4 kb in size, containing only one open reading frame (ORF) flanked with 5’ and 3’-untranslated regions (UTR). The polyprotein contains the P1, P2, and P3 regions encoding structural proteins VP1-VP4 and the non-structural proteins 2A-2C and 3A-3D, respectively (11). The viral protease 2A^pro^ is responsible for the release of P1. The intermediate 3CD of 3C^pro^ protease is responsible for the further cleavage of P1 into VP0, VP3, and VP1, and 3C^pro^ is responsible for the cleavage of P2 and P3 (12). Structural proteins VP0, VP1, and VP3 form a protomer, and five protomers form a pentamer. Twelve pentamers assemble into procapsid or naturally occurring empty particle (nEP) containing 60 copies of VP0, VP1, and VP3 (with a sedimentation coefficient 80S) (13), and they are produced during infection and are generally considered non-functional (14). As vRNA and 12 pentamers are packaged into a provirion (150S), the VP0 is cleaved into VP2 and VP4 to form a mature virion (150S or 160S) as an approximately 30 nm icosahedral particle (15). This process is autocatalytic and vRNA-dependent and is crucial for the production of infectious particles; however, one or two of the 60 VP0 molecules remain uncleaved (16). The provirion and virion are vRNA-containing particles and are often referred to as full particles (FP). Subsequently, the interaction of virion with an uncoating receptor in low pH in the endosome gives rise to swollen “A particle” (AP, 135S), which is an uncoating and vRNA-releasing intermediate (17). Normally, A particle expels VP4 and externalizes normally internal VP1 N-terminus, creating a gap that facilitates the expulsion of vRNA into cytoplasm, ultimately leaving an empty particle (EP or B particle, 80S) (18).

The life cycle of an enterovirus infection of cells includes attachment, entry, internalization, uncoating, replication, virion assembly, and release (19). Typically, enteroviruses initially interact with attachment and uncoating receptors on the cell or endosome membrane, which then initiates receptor-mediated endocytosis and uncoating. Uncoating receptor interaction triggers a conformational change of the virion, resulting in the release of the vRNA genome from the endosome into the cytoplasm across the membrane and the subsequent initiation of viral protein translation, RNA transcription, and replication. The nascent positive-stranded RNA either initiates a new round of replication or is encapsulated into progeny virus particles. Virions are released from the host cell either by a non-lysis mechanism involving extracellular vesicles or by cell lysis (20). The ring-shaped transmembrane protein KREMEN 1 is an attachment and uncoating receptor important for virus entry of host cells and genomic RNA delivery to the cytosol for CV-A6 (21).

Previous structure and mutagenesis research proposed a well-accepted mechanism for VP0 cleavage of *picornaviruses* (22, 23). A cation or base from vRNA interacts with the oxygen in a conserved VP0-H195 across *picornaviruses* and the nearby oxygen in the serine in the P1 position. The interaction of the pair of carbonyl oxygens hyperpolarizes these carbonyl groups, leading to nucleophilic attack and VP0 cleavage. The VP0 H195 possibly activates an adjacent water molecule as the nucleophile in the hydrolysis of the activated peptide bond, and packaged vRNA facilitates conformation change favoring the cleavage. Kingston et al. further identified well-conserved VP0 E096 and nearby W107, R081, and Y078 playing important roles in this process (24).

However, there was evidence that residues, located at the outside capsid surface of VP2 and VP1 and conserved only among serotypes of enteroviruses such as PV and EV-A71, regulated the efficiency of VP0 cleavage (25). The mutations were tolerable and did not abolish the cleavage completely, though the reasons were not clear.

In this study, a pair of CV-A6 strains with only a few substitutes in sequences showed differences in growth ability and virulence in cells. Mutagenesis analysis of mutants mapped a single amino acid mutation at VP1-143, which was responsible for the efficiency differences of VP0 cleavage. The results support a proposal that residues in structural proteins, located on the outside capsid surface and far away from the scissile bond, regulated the VP0 cleavage and conferred different phenotypes. These alive or viable mutants may be more relevant to the development of vaccines and anti-viral reagents.

## RESULTS

### Distinct differences in proliferation and virulence in cells between two CV-A6 strains

**Fig 1:**
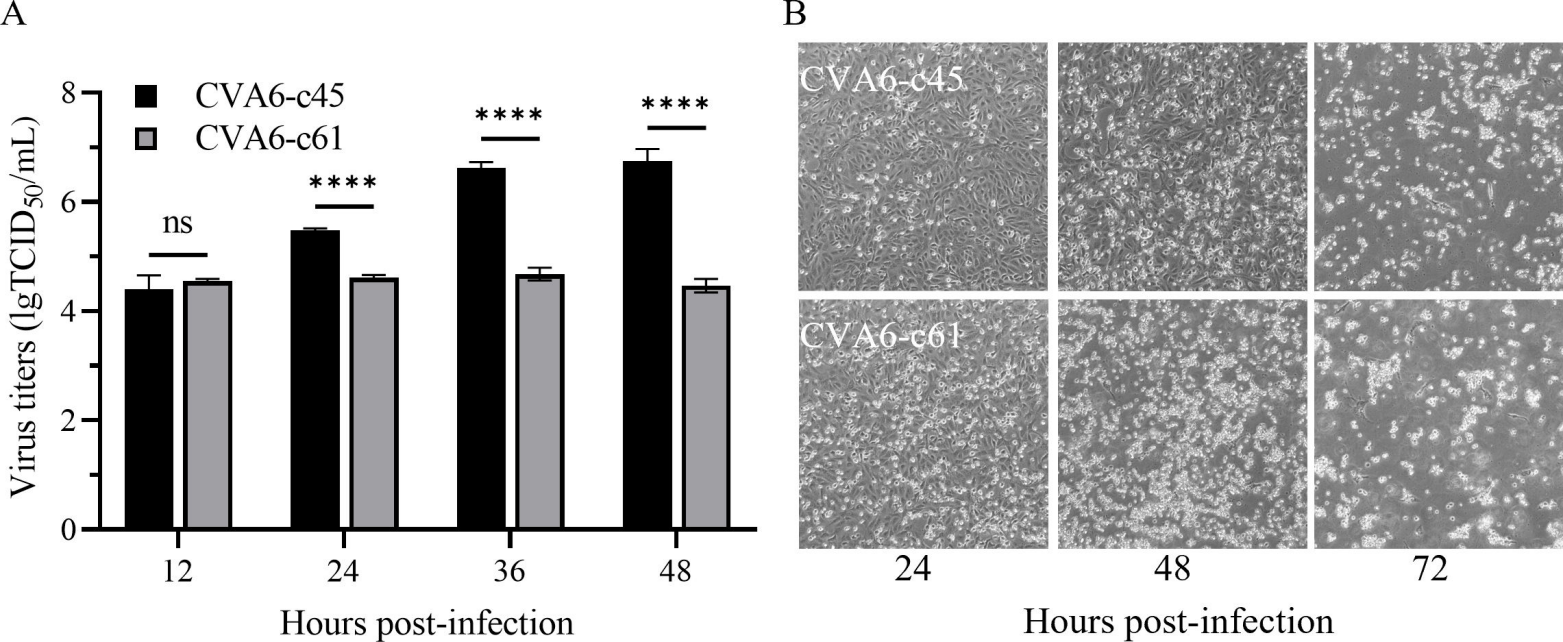
Proliferation characteristics of CVA6-c45 and CVA6-c61. (**A**) Growth ability of CVA6-c45 and CVA6-c61 in Vero cells. The viruses were inoculated in Vero cells at an MOI of 0.001, and the supernatants were collected at 12, 24, 36, and 48 h and titrated in Vero cells. The titers were averaged over three experiments, and the error bars indicated the standard deviation. (**B**) The CPE of CVA6-c45- and CVA6-c61-infected Vero cells at an MOI of 0.001 was observed at 24, 48, and 72 h post-infection. ns, not significant, *P* > 0.05; ****, *P* < 0.0001.

A CV-A6 strain, CVA6-3415/XY China/2017, was isolated in RD cells, which did not grow in Vero cells. The isolate was passaged for 18 generations in RD cells and then adapted to grow in Vero cells for 45 generations (CVA6-3415-R18V45). The later generations showed higher proliferation ability than earlier generations in Vero cells. To investigate the mechanism of phenotype changes in adaptation, plaque purification was performed using the CVA6-3415-R18V45 stock. Two clones, CV-A6-c45 and CV-A6-c61 (No. 45 and 61), showed distinctive proliferation characteristics. As shown in Fig. 1A, the titers of CVA6-c45 were more than 100-fold higher than those of CVA6-c61 within 24–48 h post-infection (*P* < 0.0001) when cells were infected at the same multiplicity of infection (MOI). To reach approximately 90% of cytopathic effect (CPE), it took 48 and 72 h at least, respectively, for CVA6-c61 (fast growth) and CVA6-c45 (slow growth) (Fig. 1B). CVA6-c45 grew to higher titer at the expense of longer incubation times, whereas CV-A6-c61 killed the cells quickly with lower titer, suggesting their different virulence *in vitro*.

The full-length sequences of the two strains were then compared, revealing three non-synonymous mutations between CVA6-c45 and CVA6-c61 (Table 1). They are threonine (T) to alanine (A) at position 139 in the VP2 (T2139A, the first number indicating the structural protein VP1 to VP3 and the rest, the amino acid site in the protein), lysine (K) to arginine (R) at position 179 in the VP3 (K3179R), and arginine (R) to glycine (G) at position 143 in the VP1 (G1143R). In addition, one nucleotide mutation and three synonymous mutations occurred at the 5′-UTR, VP1, 2C, and 3D, respectively. These mutations might contribute to the phenotype changes of high/low titer, fast-slow growth, and virulence in Vero cells.

**TABLE 1:** Nucleotide (Nt) and amino acid (Aa) differences in genomes between CVA6-c45 and CVA6-c61

Position	5′-UTR	VP2	VP3	VP1		2C	3D
Nt/Aa	689	1370/139	2259/179	2,701	2870/143	4882	6571
CVA6-c45	T	A/T	A/K	A	A/R	T	T
CVA6-c61	C	G/A	G/R	G	G/G	C	C

### The amino acid 143 of VP1 determines the distinct proliferation capacities of the two strains

**Fig 2:**
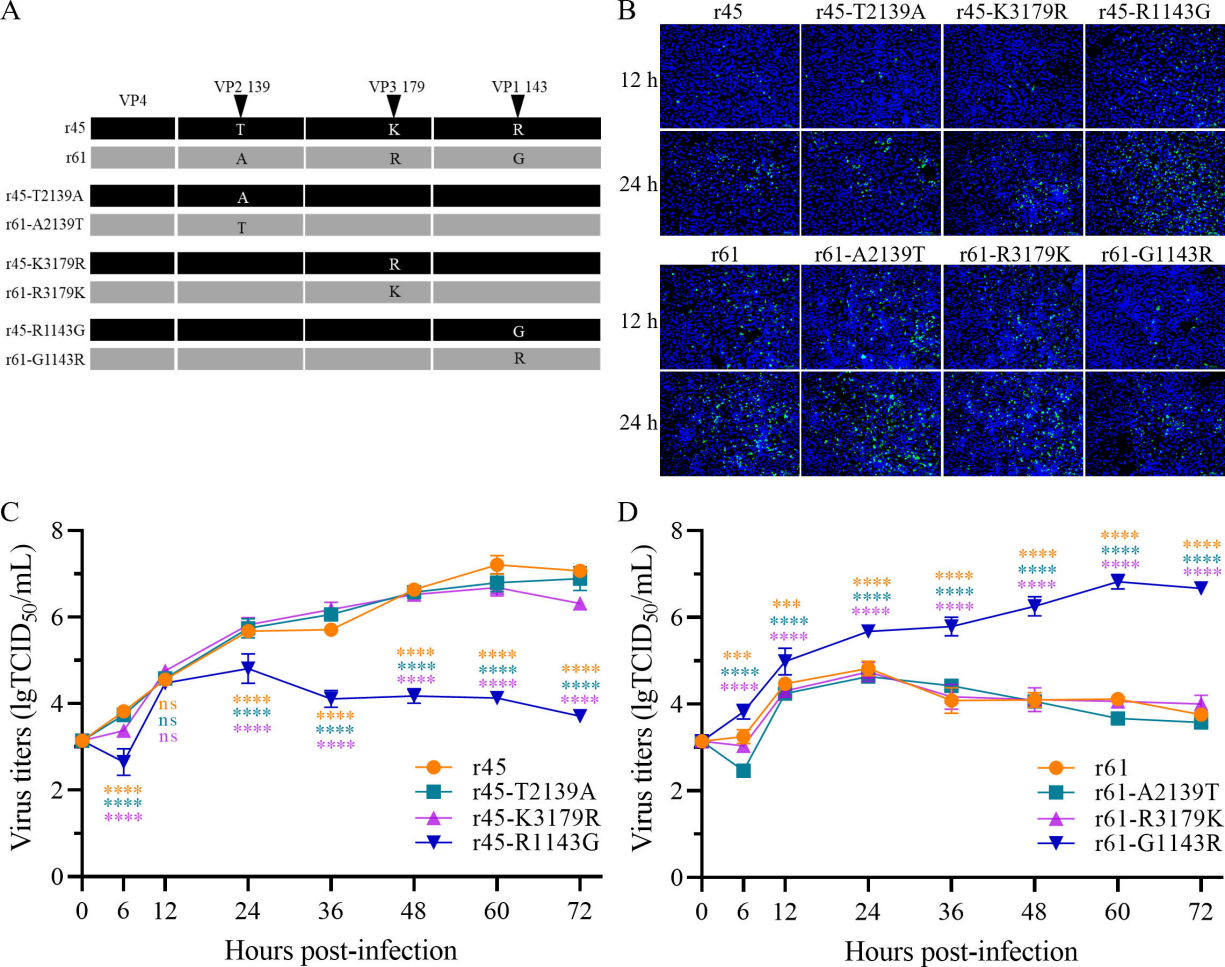
Critical role of amino acid 143 of VP1 in infectivity of CV-A6. (**A**) Schematic representation of the site-directed mutagenesis of CVA6-c45 and CVA6-c61. (**B**) Infectivity of the rescued recombinants. Vero cells were infected with each rescued strain at an MOI of 0.001, and the cells were fixed 12 and 24 h post-infection. The indirect immunofluorescence assay (IFA) was then performed using anti-CV-A6 and fluorescent secondary antibodies, and the nuclei were labeled with DAPI. (C and D) Growth curves of parental r45, r61, and their single-amino-acid mutants. Each strain was inoculated in Vero cells at an MOI of 0.001, and supernatants were collected at 6, 12, 24, 36, 48, 60, and 72 h post-infection and titrated in Vero cells. The titers represent the mean of three experiments, and the error bars indicate the standard deviation. ns, not significant, *P* > 0.05; ***, *P* < 0.001 and ****, *P* < 0.0001.

To identify the critical residues that regulate the different proliferation abilities of CVA6-c45 and CVA6-c61 in cells, the full-length infectious cDNA clones of CVA6-c45 and CVA6-c61 (abbreviated to r45 and r61) were constructed. As structural proteins of enteroviruses play important roles in cell entry, protein process, particle assemble/disassemble, and virulence, we first concentrated on the investigation of the relationship between the three non-synonymous mutations and proliferation characteristics. Based on these two parental cDNA clones, site-directed mutagenesis was performed to exchange the three amino acid residues in structural proteins VP2, VP3, and VP1 of r45 and r61 to generate six single-amino-acid mutated recombinants (Fig. 2A). The complete sequences of the recombinants were confirmed by Sanger sequencing, and the titration assay was conducted in cells.

The two parental recombinants and six single-amino-acid mutants were able to cause CPE and were able to synthesize viral proteins (detected by indirect immunofluorescence assay [IFA], more objective visualization of CPE, and virulence) following inoculation in Vero cells (Fig. 2B). At 12 and 24 h post-infection, both the amino acid mutation at position 139 of the VP2 (r45-T2139A and r61-A2139T) and the amino acid mutation at position 179 of the VP3 (r45-K3179R and r61-R3179K) exhibited fluorescence intensities comparable with those of the two parental recombinants (r45 and r61). However, at the same time post-infection, the fluorescence intensity observed for the amino acid mutation 143 of the VP1 (r45-R1143G) was markedly elevated relative to that of r45 (carrying 1143R). In contrast, the amino acid mutation 143 of the VP1 (r61-G1143R) was observed to exhibit a significantly reduced fluorescence intensity compared with r61 (carrying 1143G). The results suggested that 143G-type viruses grew fast, and the 143R-type viruses grew slowly. The mutations at the other two positions did not affect fast/slow growth ability.

The proliferation abilities of these viruses in Vero cells were assessed by comparing their growth curves. The results showed that after the mutation of the arginine at 143 of the VP1 in r45 to glycine, the highest viral titer produced by r45-R1143G was significantly reduced from 7.21 lgTCID_50_/mL in the parental r45 to 4.81 lgTCID_50_/mL (Fig. 2C). Conversely, the mutation of the r61 from glycine to arginine markedly enhanced proliferation, leading to an increase in the highest viral titer from 4.42 lgTCID_50_/mL (r61) to 6.82 lgTCID_50_/mL (r61-G1143R) (Fig. 2D). Obviously, mutating either amino acid 139 of the VP2 or amino acid 179 of the VP3 did not affect the viral titers of the recombinants derived from the two parental viruses at all (Fig. 2C and D). These results indicate that the difference in proliferation abilities between r45 and r61 is largely dependent on the amino acid at position 143 of the VP1. The amino acid at position 143 may play an important role in regulating the virus proliferation. Arginine at this position significantly favors virus propagation compared with glycine. The results also suggest that the other four nucleotide mutations at 5′-UTR and VP1 and 2C and 3D (synonymous mutations) may not associate with proliferation ability, if any, as r45-R1143G and r61-G1143R carry different nucleotides at these three positions (Table 1, T, T, T vs C, C, C, respectively).

### Role of amino acid 143 of VP1 in plaque size, virulence, rates of vRNA replication, and protein synthesis in cells

**Fig 3:**
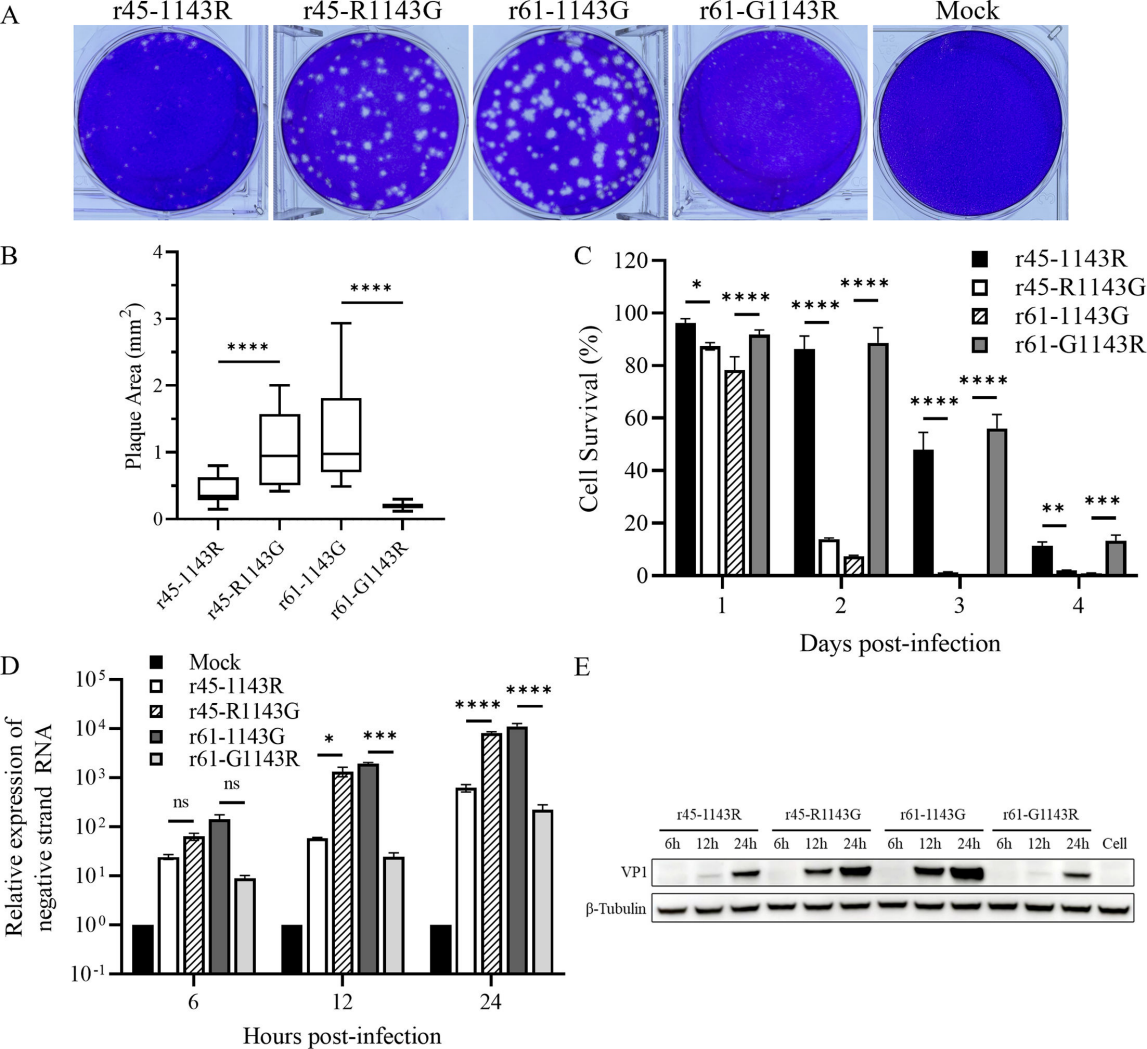
Proliferation characteristics of r45-1143R, r45-R1143G, r61-1143G, and r61-G1143R. (**A**) Representative images of r45-1143R, r45-R1143G, r61-1143G, r61-G1143R plaques, and Mock. Four days post-infection, Vero cells were fixed and stained with 1% crystal violet. (**B**) A comparison of the average plaque (*n* = 20) sizes of r45-1143R, r45-R1143G, r61-1143G, and r61-G1143R. The area of plaque was quantified using ImageJ software. (**C**) The survival of Vero cells infected with r45-1143R, r45-R1143G, r61-1143G, and r61-G1143R. (D and E) The relative expression levels of viral negative-strand RNAs and viral proteins at 24 h post-infection. Vero cells were infected with r45-1143R, r45-R1143G, r61-1143G, and r61-G1143R at an MOI of 0.001 or mock-infected. The cells were harvested at 6, 12, and 24 h, and RT-PCR and western blotting were conducted. Antibody against the viral capsid protein VP1 was used in western blotting. Anti-β-tubulin antibody was used as a loading control. Cell, mock-infected Vero cells. ns, not significant, *P* > 0.05; *, 0.01 ≤ *P* < .05; **, *P* < 0.01; ***, *P* < 0.001 and ****, *P* < 0.0001.

To investigate the roles of mutations at position 143 of the VP1 protein in virus proliferation, a comparison of the plaque size, cell lysis, vRNA, and viral protein synthesis among the parental r45-1143R (r45), r61-1143G (r61), and their single-amino-acid mutants r45-R1143G and r61-G1143R was performed. The plaque morphology showed that r45-R1143G and r61-1143G (143G-type viruses) produced significantly larger plaques than r61-G1143R and r45-1143R (143R-type viruses) did (Fig. 3A and B, *P* < 0.0001). This finding indicated that the alteration of amino acid 143 of the VP1 from glycine to arginine resulted in a reduction in plaque size and a decrease in the capacity to infect neighboring cells. In other words, the 143G type viruses spread between cells more efficiently than 143R type viruses where direct cell-to-cell virus transition occurred in the condition of plaque assays under agarose overlay. Furthermore, the cell-viability analysis, a quantitative assay of cell death and virulence, showed that at the same MOI, capacities of r45-1143R and r61-G1143R to lysis cells were markedly weaker than those of r45-R1143G and r61-1143G. As illustrated, following inoculation with r45-1143R and r61-G1143R, over 90% of Vero cells underwent lysis and death within 4 days (Fig. 3C). In contrast, equal amounts of r45-R1143G and r61-1143G only required 2 days to achieve over 90% of cell lysis (Fig. 3C). This is consistent with the growth kinetics of these strains (Fig. 2C and D), as r45-R1143G and r61 (1143G) reached peak titers at 24 h post-infection, whereas the maximum viral titers of r45 (1143R) and r61-G1143R were detected at 60 h post-infection when the most cells died.

Next, we investigated the impact of amino acids of the VP1-143 on the rates of vRNA replication and viral protein synthesis within the first 24 h post-infection by using RT-PCR and western blotting. No significant difference was observed in the relative expression capacities of the viral negative-stranded RNA of r45-1143R, r45-R1143G, r61-1143G, and r61-G1143R at 6 h post-infection. However, the relative expression capacity of the viral negative-stranded RNA of r45-R1143G and r61-1143G was significantly higher than those of r45-1143R and r61-G1143R at 12 and 24 h post-infection (Fig. 3D), which is consistent with the VP1 expression levels of the four viruses (Fig. 3E). Obviously, higher levels of vRNA and protein of 143G-type viruses were detected, compared with those of 143R-type viruses (Fig. 3E).

Taken together, these results suggest that the fast-growth 143G-type viruses spread or enter cells more efficiently and accumulate vRNAs and viral proteins more quickly than the slow-growth 143R-type viruses. A mutation in the VP1 capsid protein conferred the fast-growth phenotype by killing cells quickly, instead of mutations in CV-A6 polymerase or other replication and translation complex components. These results explain why 143G-type viruses are more virulent than 143R-type viruses in Vero cells.

### Amino acid 143 of VP1 regulates the virion maturation but not encapsidation

**Fig 4:**
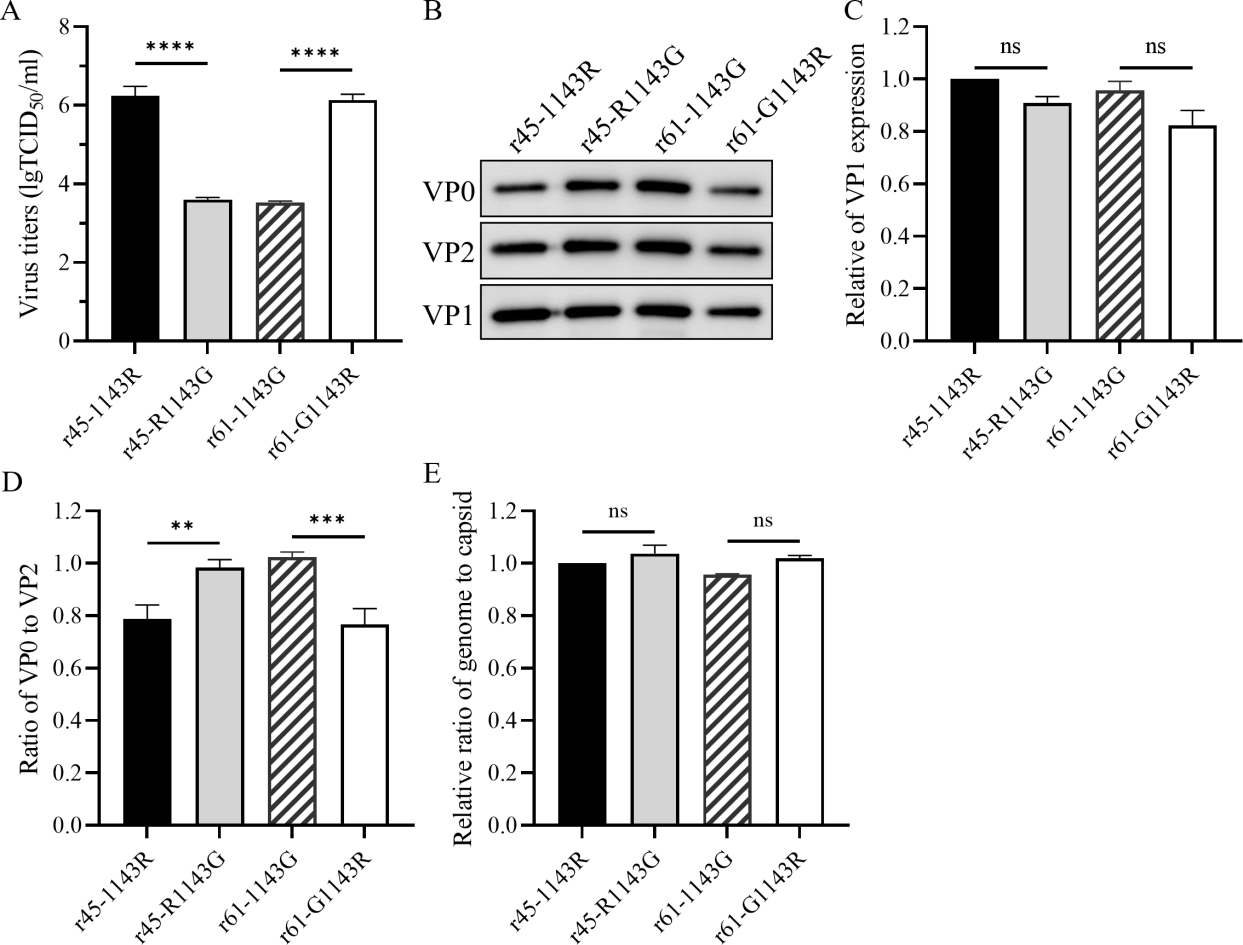
Amino acid 143 of VP1 regulating the cleavage efficiency of VP0. (**A**) Propagation of r45-1143R, r45-R1143G, r61-1143G, and r61-G1143R. Vero cells in 10-layer cell factories were infected with each of the four viruses at an MOI of 0.001. Viruses were harvested when CPEs were reached to 95%. Total viral particles from each strain were obtained by ultracentrifugation through a 20% sucrose cushion. Particles were adjusted to a concentration of 200 ng/100 µL and titrated in Vero cells. The titers were averaged across three independent replicates with error bars representing the standard deviation. (**B**) Protein profiles of total virus particles of r45-1143R, r45-R1143G, r61-1143G, and r61-G1143R. Equal volumes (1 µL) of the samples were subjected to immunoblotting using anti-CV-A6 VP0/VP2 and VP1 rabbit antibodies. (**C**) Relative expression levels of VP1 in r45-1143R, r45-R1143G, r61-1143G, and r61-G1143R. Grayscale values of each band were quantified using ImageJ software and normalized relatively to r45-1143R. (**D**) Effect of amino acid 143 of VP1 on the cleavage efficiency of VP0. Grayscale values of each band were quantified using ImageJ software. The cleavage efficiency of VP0 was expressed as the ratio of VP0 to VP2. (**E**) The impact of amino acid 143 of VP1 on vRNA packaging efficiency. The vRNA copy numbers were quantified by RT-PCR, and capsid protein content was measured using the bicinchoninic acid (BCA) assay. The packaging efficiency was calculated as the ratio of RNA copy number to the amount of capsid protein, and the ratio was normalized relative to that of r45-1143R. ns, not significant, *P* > 0.05; *, 0.01 ≤ *P* < .05; **, *P* < 0.01; ***, *P* < 0.001 and ****, *P* < 0.0001.

The objective of the subsequent investigation is to gain further insight into the mechanism of amino acid 143 of VP1 on the proliferation ability of viruses in cells. The four strains were inoculated in Vero cells at the same MOI, and viruses were harvested when 95% of the cells showed obvious CPE. Total virus particles, including vRNA-containing full and A particles, and empty particles (11) of the four strains were obtained by ultracentrifugation on a 20% sucrose cushion. The viral particle samples were resuspended to an equal volume, as required for subsequent comparison. First, the virus particles of four strains were diluted to 200 ng/100 µL for titration. As expected, the viral titers of r45-1143R and r61-G1143R were over 100-fold higher than those of r45-R1143G and r61-1143G when all strains reached their peak hours of propagation (Fig. 4A). Then, the equal volume of samples was taken for immunoblotting assay of VP1, representing the virus particle (Fig. 4B). Notably, the results indicated no significant difference in the amounts of total viral proteins between r45-1143R and r45-R1143G or between r61-1143G and r61-G1143R. The difference in VP1 levels is less than 20% among the four viruses. These findings suggest that the amino acid at 143 of VP1 does not significantly influence the proliferation capacity of the viruses by regulating the yields of total viral particles. This observation led us to detect the ratio of infectious (VP0 cleaved) and non-infectious (VP0 not cleaved) viral particles of the four recombinants.

The differences in VP0 cleavage efficiency between the strains were checked by using antibodies that recognized both VP0 and VP2. The ratio of VP0 to VP2 proteins was calculated, reflecting the relative amounts of infectious virion (VP0 cleaved) and non-infectious provirion (VP0 not cleaved). As illustrated in Fig. 4D, the mature, infectious virions of r45-1143R and r61-G1143R were significantly higher (lower ratio of VP0 to VP2) than those of r45-R1143G and r61-1143G (higher ratio of VP0 to VP2). Subsequently, the viral genomic RNA and capsid proteins of the viral particle were quantified to calculate the ratio of vRNA to capsid protein (Fig. 4E), reflecting vRNA packaging efficiency. It was found that there was no significant difference in the RNA packaging efficiency of the four recombinants, no matter which amino acid residues the recombinants carried at the VP1 position 143.

### Amino acid 143 of VP1 regulating cleavage efficiency of the provirion VP0

**Fig 5:**
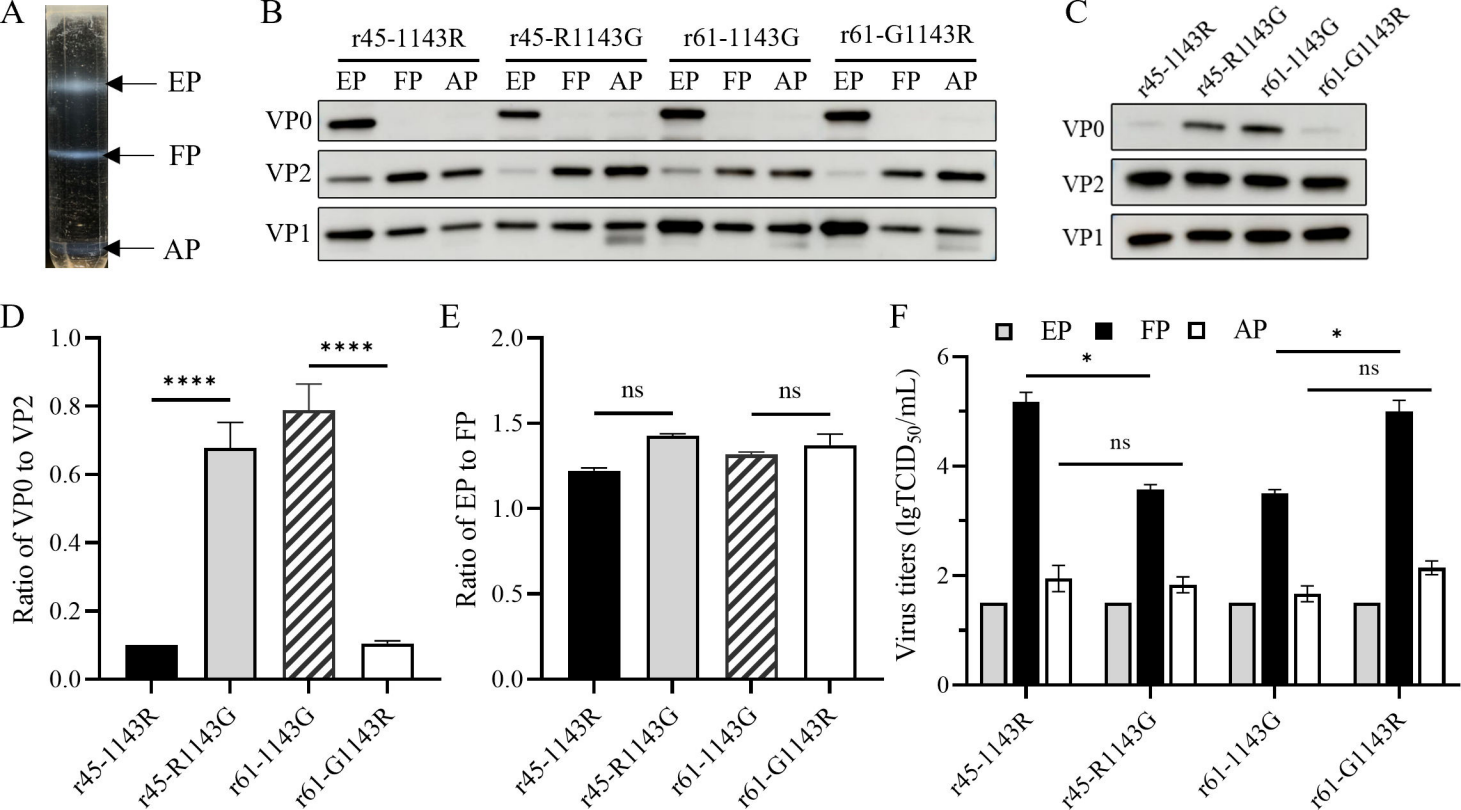
Amino acid 143 of VP1 regulating cleavage of the VP0 in provirion. (**A**) A representative CsCl gradient ultracentrifugation tube and virus fractions. (**B**) Western blotting analysis of EP, FP, and AP. The EP, FP, and AP of r45-1143R, r45-R1143G, r61-1143G, and r61-G1143R purified through CsCl density gradient centrifugation were subjected to immunoblotting with anti-CV-A6 VP0/VP2 and VP1 rabbit antibodies. (**C**) Excess FP samples were subjected to immunoblotting with anti-CV-A6 VP0/VP2 and VP1 rabbit antibodies to detect traces of VP0 in FP fractions. (**D**) Gray-scale values of each band in (**C**) were determined using ImageJ, and VP0 cleavage efficiency was expressed as the ratio of VP0 to VP2. The relative ratio was normalized to that of r45-1143R. (**E**) The capsid protein content of all EP and FP fractions was quantified using the BCA assay, and the ratio of EP to FP was subsequently calculated. (**F**) Infectivity of the EP, FP, and AP of the recombinants. Each fraction of r45-1143R, r45-R1143G, r61-1143G, and r61-G1143R was diluted to 200 ng/100 µL and titrated in Vero cells. The titers are averaged over three replicates, and the standard deviations are indicated by error bars. ns, not significant, *P* > 0.05; **, *P* < 0.01 and ****, *P* < 0.0001.

To further verify the aforementioned conclusions of VP0 cleavage efficiency, we employed CsCl density gradient centrifugation to purify viral particles from the four strains for analysis. The three fractions, from the top to the bottom, were collected (Fig. 5A). First, western blotting was conducted to analyze the capsid protein profiles in each fraction (Fig. 5B). The results proved that the three main fractions were the empty particle (EP), full particle (FP), and altered particle (AP). EP was found to be devoid of vRNA, whereas both FP and AP were found to contain vRNA (data not shown). When rabbit anti-VP1 polyclonal antibody was used, a smaller band below the intact VP1 was detected in AP fractions, indicating that the VP1 N-termini was exposed outside of the AP and was sensitive to cellular proteases as we reported previously on CV-A5 (26–28).

To examine the effect of amino acid 143 of VP1 on the efficiency of VP0 cleavage to form mature virion, the FP fractions, in which provirion and virion were inseparable in CsCl gradients, of the four strains were analyzed using western blotting with excessive samples and longer exposure (Fig. 5C). The ratio of VP0 to VP2 was determined by a quantitative assay to assess the relative abundance of VP0 and VP2 in FP fractions (Fig. 5D). The results showed that r45-1143R and r61-G1143R had a significantly lower VP0/VP2 ratio than r45-R1143G and r61-1143G, decreasing by 6.7-fold and 7.8-fold (*P* < 0.0001). Glycine-to-arginine mutation resulted in more virions and less provirions. Another explanation is that virions in FP fractions of 143R-type strains contain lesser uncleaved VP0 (16). Nonetheless, in both circumstances, the results directly confirmed that the VP1-143 residues regulated the VP0 cleavage in provirion and were consistent with the results obtained using total numbers of different particles (Fig. 4D). Subsequently, the ratio of EP to FP was calculated for each of the four strains, and no significant difference was found between G- and R-type viruses, indicating that the mutation did not influence encapsidation of vRNA (Fig. 5E).

The titration results of 200 ng of each fraction (Fig. 5F) indicated that no viral titers were detected in all three EP fractions of each of the four viruses (below the lower limit of detection of 1.50 lgTCID_50_/mL, assign 1.50 for graphing). The viral titers observed for all AP fractions were very low, with values of 1.95, 1.83, 1.67, and 2.14 lgTCID_50_/mL, respectively. It was understandable considering that most of the APs might be the later form characterized with extended/degraded VP1 and lost VP4 as we reported previously on CV-A5 (Fig. 5B) (26). The FP fractions of r45-1143R and r61-G1143R exhibited virus titers of up to 5.18 lgTCID_50_/mL and 5.00 lgTCID_50_/mL. In comparison, the virus titers of the FP fractions of r45-R1143G and r61-1143G were significantly lower at 3.58 lgTCID_50_/mL and 3.50 lgTCID_50_/mL, respectively. The results suggest that in FP fractions, there are two components: infectious virion and non-infectious provirion. Importantly, the result indicated that in the same number of vRNA-containing FPs, r45-R1143G and r61-1143G contained a lesser number of infectious, mature particles than those of r45-1143R and r61-G1143R. The mutation of amino acid 143 of VP1 from arginine to glycine decreased the cleavage efficiency of VP0 in provirion, which resulted in an increase in the proportion of non-infectious provirion to infectious virion, likely leading to the accumulation of provirion and the low titer.

## DISCUSSION

Cleavage of VP0 into VP4 and VP2 is stabilizing maturation cleavage of *picornaviruses* as it is required for conformational transitions of non-infectious provirion to infectious virion. The VP0 cleavage is a vRNA-involved, autocatalytic process, although the packaging of vRNA is required but not sufficient to lead to cleavage. Previous research proposed a well-accepted mechanism of VP0 cleavage and *picornaviruses* virion assembly (22, 23). Amino acid residue at VP2 H195, closely located to the VP4/VP2 scissile bond in provirion and was conserved in the structural protein across VP0 cleavage *picornaviruses*, participated directly in the cleavage. Mutation of this residue to the other four residues completely abolished the cleavage, and the mutants were fatal and unable to pass in cells. Kingston et al*.* discovered that a well-conserved VP0 E096 (VP2-E27) in VP0 of all enterovirus types has an essential role in VP0 cleavage independent of RNA encapsidation (24). The E096 is located at the scissile region, interacts with nearby W107 and VP0 R081, Y078 of VP0, and is responsible for conformation change, vRNA encapsidation, and VP0 cleavage. The mutation from E to A residue abolished the VP0 cleavage and was fatal.

Our work supports another mechanism that other amino acid residues on a structural protein, not positioned near the cleavage site, exposed on the outside surface of the capsid, and not well-conserved across *picornaviruses*, regulate the efficiency of the VP0 cleavage. In our research, the residue VP1 143 of a CV-A6 is exposed at the outside DE loop, conserved only within serotype CV-A6, and is distant to the cleavage site, being 77.2 Å away from the VP0 cleavage site. The strains with glycine or arginine at this position impaired or enhanced the efficiency of the VP0 cleavage, respectively. The mutants are not lethal mutants and still can pass in cells. The proposal is based on accumulated evidence. An early study by Compton et al. found that a conditional mutant of PV (VP2 BC loop, R76Q) showed a defect in VP0 cleavage when it was grown at 39°C but had a near WT phenotype when it was cultured at 32^°^C (29). The VP2 R76Q is located at the BC loop and is also exposed on the outer surface of the capsid. The regulation mechanism of the VP0 cleavage by a distant residue remains unclear. Interestingly, Zhang et al. reported that an alanine residue at the VP1 107 position at βC near the BC loop, distant from the scissile boundary of VP0, was conserved only among the EV-A71 but not across enteroviruses and increased the efficiency of the maturation cleavage (25), compared with a mutant. Furthermore, seven single-amino-acid mutants from alanine to different electrostatic potential residues at this position did not completely abolish the VP0 cleavage but decreased the efficiency of VP0 cleavage. The authors propose that a flexible conformation of the BC and EF loops contributes to the efficient virion maturation and propagation of EV-A71 by molecular dynamics (MD) simulation and hydrogen-bond network analysis. Our results provide evidence that a non-conserved residue across enterovirus types and far away from the cleavage site regulates VP0 cleavage and proliferation. CV-A6 strain in this study may provide a high ratio of accumulated provirion, which is difficult to obtain, for structural analysis, which is required to study the precise mechanism and support our proposal.

Historically, non-EV-A71 and non-CV-A16 coxsackieviruses in species A are difficult to cultivate in cells (30, 31). In our previous study, only 10 CV-A6 were isolated in RD cells but not in Vero cells from more than 2,000 CV-A6 vRNA-positive clinical samples (9). CPE of earlier RD and Vero-adapted passages is difficult to observe, and earlier Vero-adapted passages did not form plaques. Non-lytic infection and low efficiency of VP0 cleavage of CV-A6 may be the two reasons for this phenomenon. Büttner et al. analyzed the structures of the CV-A6 (prototype Gdula) infectious virion, altered particle, and non-infectious empty capsid. It was found that the ratio of purified infectious units to virion is 1–500 based on counting the particles using cryo-ET (11). The low ratio of infectious vRNA-containing particles might be caused by a variety of reasons, such as low efficiency of VP0 cleavage. Sequence alignment around VP1-143 was performed based on 2,837 CV-A6 VP1 available in GeneBank. The frequencies for glycine and arginine are 98.943% and 0.987%, respectively, explaining the difficulty of CV-A6 isolation in cells. The low efficiency of VP0 cleavage is an infection barrier for CV-A6 in cells. A structural study by Xu et al*.* found that the majority of particles of a CV-A6 Taiwan strain are A particles, demonstrating the dependence of the ratio of different particles on different CV-A6 subgenotypes (17).

In conclusion, a single mutation from glycine to arginine at VP1 143 increased the efficiency of VP0 cleavage into VP4 and VP2 and the ratio of infectious virion but decreased virulence *in vitro* in Vero cells. Inefficiency of VP0 cleavage resulted in the accumulation of provirion of CV-A6. The data provide new evidence supporting a hypothesis that the residues at structural proteins conserved only within an enterovirus serotype/genotype regulate the VP0 mature cleavage. This hypothesis is a complement to the previous one where evolutionarily conserved residues across *picornaviruses* and structurally close to the VP0 cleavage site are directly involved in the cleavage. The regulation results in “alive” mutants rendering different phenotypes relevant to infectivity, virulence, and fitness of enteroviruses. Next, we plan to focus our research on the pathogenicity and *in vivo* passaging experiments of these mutants. *In vivo* passaging can help confirm the relevance of these mutations in viral transmission and pathogenicity, providing a comprehensive understanding of the impact of viral adaptation in more complex biological environments.

## MATERIALS AND METHODS

### Cells and viruses

African green monkey kidney (Vero) cells, human rhabdomyosarcoma (RD) cells, and Golden hamster kidney cells containing T7 RNA polymerase (BSR-T7/5) cells were maintained in our laboratory. Cells were cultured in DMEM (Thermo Fisher Scientific, USA) medium supplemented with 10% neonatal bovine serum. A CV-A6 strain, designated as CVA6-3415/XY China/2017, was isolated in RD cells from a clinical specimen obtained from a child with HFMD in Xiangyang, China, in 2017 (9). Further passaging in RD cells and adapting in Vero cells were performed to obtain the plaque-purified strains in Vero cells, designated CVA6-c45 and CVA6-c61 (Clones 45 and 61 derived from the virus quasispecies of CVA6-3415-R18V45, passaged in RD and Vero cells 18 and 45 generations, respectively).

### Construction of full-length infectious cDNA cloning and mutants

The complete genome sequences for the CVA6-c45 and CVA6-c61 strains were obtained using reverse transcription and polymerase chain reaction (RT-PCR). The full-length sequences were divided into two contiguous fragments (A: 5'UTR-VP1; B: 2A-polyA) and subsequently cloned into the pBR322 vector by homologous recombination. The resulting plasmids were designated as pBR322-CVA6-c45 and pBR322-CVA6-c61, respectively. These two plasmids were employed to generate the full-length cDNA clones of mutants. All cDNA clones with distinct point mutations were prepared using specific primer pairs by PCR-targeted mutagenesis.

### Virus rescue and passage

BSR-T7/5 cells were cultured in 12-well plates until 50% confluence was reached. Then, 1 µg of the plasmid was transfected into the BSR-T7/5 cells using Lipofectamine 3000, following the kit instructions. After 48 h of incubation, viruses in the cells and supernatants were collected by freeze-thawing four times, and cellular debris was removed by centrifugation at 3,900 × *g* for 10 min. The Vero cells were infected with the supernatants and monitored daily for CPE. The rescued virus was then propagated in Vero cells for three consecutive generations, and viral titers were determined and used for subsequent analysis.

### Growth kinetics curve

Each virus infected Vero cells in 12-well plates at an MOI of 0.001. Following an hour of adsorption, the cells were washed three times with PBS, and then DMEM maintenance solution was added. Supernatants were collected at the designated time points following infection, and viral titers were determined through the 50% tissue culture infective dose (TCID_50_) method.

### Plaque assay

Vero cells were seeded in 6-well plates and allowed to grow until they reached 90% confluency. Then, 500 µL of serial 10-fold gradient diluted virus samples were seeded into the 6-well plates and incubated at 37°C for 1 h. Afterward, the cells were washed three times with PBS, and then, 2 mL of a DMEM medium containing 1% low melting agarose was added. The cells were incubated at 37°C for 5 days, after which they were fixed in 4% paraformaldehyde fixative for 1 h at room temperature, and then, the cells were stained with a 1% crystal violet solution.

### Cell proliferation assay

Vero cells were seeded in 96-well plates, and when the cells grew to 90%, each strain infected the cells at an MOI of 0.001. Three parallel samples, including a blank control group in each group; then, 10 µL of CCK-8 detection solution was added to each well at the designated time points, and the cells were incubated at 37°C for 1 h. The absorbance was detected at 450 nm with an enzyme marker, and the cell survival rate was calculated.

### Antibody and western blotting assays

Rabbit polyclonal antibodies recognizing the CV-A6 VP0/VP2 and VP1 capsid proteins were prepared for this experiment. The anti-β-tubulin mouse monoclonal antibody (YAZHUA, China), HRP-conjugated AffiniPure goat anti-rabbit IgG(H + L) (BOSTER, China), and HRP-conjugated AffiniPure goat anti-mouse IgG(H + L) (BOSTER, China) were purchased. Infected cells and concentrated virus samples were lysed in pre-cooled RIPA buffer, and protein samples were separated by 12% SDS-PAGE. Proteins were transferred to NC membranes and blocked with 5% skim milk for 1 h at room temperature, followed by incubation with diluted primary antibodies, including anti-CV-A6 VP0/VP2 rabbit antibody (1:5,000 diluted), anti-CV-A6 VP1 rabbit antibody (1:5,000 diluted), or anti-β-tubulin antibody (1:5,000 diluted), for 1 h at room temperature. Subsequently, the membranes were washed three times with PBS containing 0.1% Tween 20 (PBST) and incubated for 1 h at room temperature with a 1:10,000 dilution of HRP-conjugated secondary antibody. After three washes with PBST, the signal was detected using ECL chemiluminescence reagent (Millipore).

### Indirect immunofluorescence assay (IFA)

Vero cells were inoculated into 12-well plates and cultured until 90% confluence was reached. Cells were infected with the viruses and incubated (cultured) until significant CPE appeared. Cells were washed three times with PBS, fixed with 4% paraformaldehyde in PBS for one hour at room temperature, and permeabilized with 0.5% Triton X-100 in PBS for 15 min at room temperature. After blocking with 1% BSA in PBS for 30 min at room temperature, cells were incubated with anti-CV-A6 rabbit antibody (1:200 dilution) for 1 h at room temperature. After washing, Alexa Fluor 488-labeled goat anti-rabbit IgG (H + L) (1:500 dilution) (Beyotime, China) was added and incubated at room temperature for 1 h. After further washing, DAPI (1:1000 dilution) was added and incubated at room temperature for 10 min. Cells were washed three times with PBS and photographed under a fluorescence microscope.

### Real-time RT-PCR assays

Vero cells were seeded in 12-well plates until 90% confluence. Cells were infected with a virus at an MOI of 0.001. Total RNA was extracted using the FastPure Cell/Tissue Total RNA Isolation Kit V2 (Vazyme, China) at the indicated time points post-infection. One microgram of total RNA was used to synthesize total cellular cDNA using oligo(dT) primers, and another 1 µg of total RNA was used to synthesize viral negative-strand cDNA with primers designed according to the complementary strand of CV-A6 (5′-TTAAAACAGCTTGTGGGGTT-3′). All reverse transcription reactions were performed using HiScript II Q RT Super Mix for qPCR (Vazyme, China). Amplification was performed using ChamQ Universal SYBR qPCR Master Mix (Vazyme, China) with a thermal cycling program of 95°C for 30 s, followed by 40 cycles of 95°C for 10 s, and 60°C for 30 s. Relative mRNA expression levels were normalized to β-actin mRNA. Relative transcript levels were analyzed by the ΔΔCt method. Primer sequences used in the experiment were listed as follows:

CVA6-VP1-F:5'-ATATTCGCAAAATTGAGTGATCCAC-3',

CVA6-VP1-R:5'-GTTATTAGGACATTGCCCATATTGC-3'.

Actin-F:5′-TGAAGTGTGACGTGGACATCCG-3′,

Actin-R:5′-GCTGTCACCTTCACCGTTCCAG-3′.

### Measurement of viral genomic RNA

vRNA was extracted from purified virus samples. Quantification of RNA was conducted using real-time PCR with the One Step TB Green PrimeScript RT-PCR Kit (Takara, Japan) and primers CVA6-VP1-F and CVA6-VP1-R. The thermal cycling program consisted of 42°C for 5 min, 95°C for 10 s, followed by 40 cycles of 95°C for 5 s and 60°C for 34 s. The plasmid containing the DNA sequence of the VP1 gene was transcribed and purified *in vitro* according to the instructions, and the RNA standard obtained was serially diluted to generate a standard curve and calculate the copy number of viral genomic RNA.

### Purification of viral particles

Cells and supernatants were collected by freeze-thawing, followed by centrifugation at 11,100 × *g* for 1 h to remove cell debris. The viral supernatant was then concentrated by ultrafiltration using a 100 kDa ultrafiltration membrane pack. Purification of the concentrate was achieved by ultracentrifugation at 96,598 × *g* for 3 h at 4°C through a 20% sucrose cushion. The resuspended virus pellet was further purified by CsCl (1.3 g/mL) density gradient ultracentrifugation at 132,716 × *g* for 20 h at 4°C. After centrifugation, a syringe was used to collect the bands in the CsCl centrifuge tube, from top to bottom, corresponding to EP, FP, and AP.

### Statistical analysis

All statistical analyses were performed using GraphPad Prism 9 software. The experimental data are presented as the mean ± standard deviation (SD) of three biological replicates. The results of different experimental groups were compared using two-way and one-way ANOVA. The levels of statistical significance were as follows: ns, not significant, *P* > 0.05; *, 0.01 ≤ *P* < .05; **, *P* < 0.01; ***, *P* < 0.001, and ****, *P* < 0.0001.

## Data Availability

The complete sequences of CVA6-c45 and CVA6-c61 have been deposited in GenBank with the accession numbers PV232302 and PV232303, respectively.
